# Temporal trend and factors associated with physical disability due to leprosyl: a time series study, Brazil, 2014-2023

**DOI:** 10.1590/S2237-96222026v35e20240899.en

**Published:** 2026-05-01

**Authors:** Everton dos Santos Araújo, Jefferson Felipe Calazans Batista, Yasmim Ingrid Nascimento Santos, Marcos Antonio Almeida-Santos

**Affiliations:** ¹ Universidade Tiradentes, Programa de Pós-Graduação em Saúde e Meio Ambiente, Aracaju, SE, Brazil

**Keywords:** Leprosy, Multibacillary, Leprosy, Tuberculoid, Mycobacterium leprae, Time Series Studies, Cross-Sectional Studies., Lepra Multibacilar, Lepra Tuberculoide, Mycobacterium leprae, Estudios de Series Temporales, Estudios Transversales.

## Abstract

**Objective:**

To analyze the temporal trend and factors associated with physical disability due to leprosy in Brazil from 2014 to 2023.

**Methods:**

Time series study subdivided into the analysis of operational indicators and factors associated with the degree of disability due to leprosy in Brazil. The data were extracted from the Notifiable Diseases Information System (*Sistema de Informação de Agravos de Notificação*, SINAN). The incidence rate of leprosy with grade 2 physical disability per 1,000,000 inhabitants was calculated. The associated factors were measured by logistic regression, which generates the odds ratio (OR), the trend by Prais- Winsten regression, and the annual percentage change.

**Results:** A total of 228,577 cases of leprosy were analyzed in Brazil between 2014 and 2023. The Central-West and North regions presented rates of 19.61/1 million and 19.12/1 million, respectively. The annual percentage change was 6.0 (95%CI 5.1; 6.9) for new cases with grade 2 disability in Brazil. The factors associated with grade 1 disability were: ≥60 years (OR 3.36; 95%CI 1.03; 3.15), tuberculoid form (OR 0.95; 95%CI 0.89; 1.04), and low education level (OR 1.50; 95%CI 1.03; 1.41). For grade 2, the associated factors were male sex (OR 1.56; 95%CI 1.02; 1.51), pregnancy (OR 1.11; 95%CI 0.86; 1.13), and multibacillary form (OR 1.99; 95%CI 1.14; 1.54).

**Conclusion:**

In summary, lower chances of presenting grade 1 or grade 2 physical disabilities were associated with female sex, tuberculoid clinical form, residence in the Southeast and South regions, and higher education level.

## Resumen

**Objetivo:**

Analizar la tendencia temporal y los factores asociados con la discapacidad física por lepra en Brasil durante el período 2014-2023.

**Métodos:**

Estudio de series temporales subdividido en el análisis de los indicadores operacionales y de los factores asociados al grado de discapacidad por lepra en Brasil. Los datos se extrajeron del Sistema de Información de Enfermedades Notificables (*Sistema de Informação de Agravos de Notificação*, SINAN). Se calculó la tasa de incidencia de lepra con discapacidad física grado 2 por 1 millón de habitantes. Los factores asociados se evaluaron mediante regresión logística, generando la razón de probabilidades (*odds ratio*, OR); la tendencia se analizó mediante la regresión de Prais-Winsten y la variación porcentual anual.

**Resultados:**

Se analizaron 228.577 casos de lepra en Brasil entre 2014 y 2023. Las regiones Centro-Oeste y Norte presentaron tasas de 19,61/1 millón y 19,12/1 millón, respectivamente. La variación porcentual anual fue de 6,0 (IC95% 5,1; 6,9) para los casos nuevos con grado 2 de discapacidad en Brasil. Los factores asociados al grado 1 de discapacidad fueron: ≥60 años (OR 3,36; IC95% 1,03; 3,15), forma tuberculoide (OR 0,95; IC95% 0,89; 1,04) y bajo nivel educativo (OR 1,50; IC95% 1,03; 1,41). Para el grado 2, los factores asociados fueron sexo masculino (OR 1,56; IC95% 1,02; 1,51), embarazo (OR 1,11; IC95% 0,86; 1,13) y forma multi­bacilar (OR 1,99; IC95% 1,14; 1,54).

**Conclusión:**

En resumen, se observaron menores probabilidades de presentar discapacidades físicas de grado 1 o 2 asociadas con el sexo femenino, la forma clínica tuberculoide, la residencia en las regiones Sudeste y Sur y un nivel educativo superior.

**Ethical aspects**

This research used public domain anonymized databases.

## Introduction 

*Mycobacterium leprae* is the causative agent of leprosy, a disease that represents a major challenge for public health in Brazil. Considered one of the main endemic diseases in the country, leprosy affects the quality of life of affected individuals and imposes high socioeconomic costs. The degree of physical disability, reflected by the presence of physical limitations in patients, is a crucial indicator to measure the impact of the disease and the effectiveness of public health interventions. Between 2014 and 2023, Brazil witnessed variations in the rates of disabilities associated with leprosy, which requires an in-depth analysis of temporal trends and factors that influence these results ^1^.

Brazil is the second country with the highest number of new cases. This situation may be associated with various factors, such as inadequate access to health services, lack of early diagnosis, and socioeconomic inequalities, which limit effective prevention and treatment ^2^. Monitoring operational indicators is essential to guide public policies and improve patients' quality of life, while strengthening support networks and coordination between different sectors is fundamental to reducing disabilities and promoting the dignity of affected individuals ^3^ ^,^ ^4^. 

Regional inequality is a determining factor for leprosy in Brazil; the North and Northeast regions, for example, concentrate high rates of incidence and physical disabilities. These data indicate a correlation between poverty, low education, and greater vulnerability to leprosy, despite investments in epidemiological surveillance strategies and awareness campaigns. However, the effectiveness of these actions remains questionable, considering the data on the persistence of physical disabilities. The lack of coordination between health policies and primary health care services may hinder the effective implementation of strategies aimed at reducing disabilities ^5^ ^,^ ^6^.

Furthermore, the performance of the health system regarding physical disability due to leprosy in Brazil over the past ten years reveals complex and interconnected challenges. Therefore, we aim to analyze the temporal trend and factors associated with physical disability due to leprosy in Brazil from 2014 to 2023.

## Methods 

Study design

An exploratory and analytical time series study with a quantitative approach, subdivided into temporal analysis of operational indicators and factors associated with the degree of disability due to leprosy in Brazil ^7^.

Setting

Public data on leprosy cases in Brazil and its macroregions from January 2014 to May 2023 were used.

Participants

Confirmed cases of leprosy in the Brazilian population were considered. Cases classified as "diagnostic error" in the "type of outcome" variable were excluded, as they were not characterized as leprosy cases.

Variables

The following variables were selected:

Sociodemographic:

Detailed age (in years);

Gender (Male; Female);

Country macroregion (North; Northeast; Southeast; South; Central-West);

Year of diagnosis (2014-2023);

Race/skin color (White; Black; Brown [Brazilian mixed race ]; Asian; Indigenous);

Education level (illiterate; incomplete elementary school; complete elementary school; incomplete high school; complete high school; incomplete higher education; complete higher education); and

Pregnant person (1st trimester; 2nd trimester; 3rd trimester; unknown pregnancy age; no; not applicable; unknown).

Clinical:

Clinical form (indeterminate; tuberculoid; borderline; lepromatous; unclassified; unknown);

Degree of disability at diagnosis (grade 0; grade 1; grade 2; not evaluated; unknown);

Operational classification (new case; transfer from the same municipality [or another state ]; transfer from another municipality [same state ]; transfer from another state; transfer from another country; recurrence; other re-entries; unknown);

Mode of detection of new case (referral; spontaneous demand; community survey; contact examination; other modes; unknown);

Bacilloscopy (positive; negative; not performed; not applicable);

Initial therapeutic regimen (PQT/PB/6 doses; PQT/MB/12 doses; other substitute regimens);

Reactional episode during treatment (type 1 reaction; type 2 reaction; type 1 and 2 reaction);

Number of skin lesions;

Number of registered contacts;

Number of contacts examined;

Number of supervised doses; and

Number of affected nerves.

The variable degree of disability at diagnosis was considered the dependent variable. This variable was expressed as "physical disability." In the aggregated data, some operational indicators of leprosy associated with the degree of disability were calculated, following the standards of the Ministry of Health, namely ^8^:

Proportion of new leprosy cases with disability grade assessed at diagnosis.

Proportion of new leprosy cases with grade 2 physical disability at diagnosis.

Incidence rate of leprosy with grade 2 disability at diagnosis per 1,000,000 inhabitants.

Proportion of new leprosy cases in the 0-14 years population with grade 2 physical disability at diagnosis

Data sources/measurement

Leprosy data were extracted from the Brazilian National Health System Information Technology Department (*Departamento de Informática do Sistema Único de Saúde -* DATASUS) through the Notifiable Health Conditions Information System (Sistema de Informação de Agravos de Notificação - SINAN) ^9^. At the same time, the resident population used for calculating indicator 3 was obtained from the 2010-2060 population projections of the Brazilian Institute of Geography and Statistics (*Instituto Brasileiro de Geografia e Estatística*, IBGE) ^10^. All data were accessed in July 2024 as follows: 

Access to the annual databases (2014-2023) of notification forms on the website of the Brazilian National Health System Information Technology Department-data in *.dbc* format.

Import of the downloaded databases into TabWin for conversion from *.dbc* to *.dbf.*

Merging of the annual databases into a single database.

Tabulation of data for operational indicators, following the Ministry of Health standards.

Export of microdata for processing in Microsoft Excel.

The research database is available online ^11^. 

Variables

The following independent variables were modified: detailed age → age group (0-14 years; 15-19 years; 20-39 years; 40-59 years; ≥60 years); education level → grouped into illiterate, elementary, high school, and higher education; input mode → all transfer categories were combined into a single group, "transfer"; pregnant person → first to third trimester + unknown age were grouped as "yes." 

In the variable "clinical form," there were 39 individuals whose group was recorded as "6," a value inconsistent with the notification form and data dictionary. Therefore, this incorrect classification was converted to missing data. For data analysis, variables with groups labeled "unknown" or "not applicable" were also converted to missing data.

Bias control

Considering the presence of missing data in the study and aiming to improve the predictive model, a multiple imputation procedure was performed based on the assumption that the losses were missing at random, a common occurrence in studies of this nature ^12^. Therefore, by including all available variables and using the predictive mean matching method, five imputation models were proposed ^13^. 

Data availability

*Aggregated data*

The temporal trend was estimated using Prais-Winsten regression. All indicators were transformed to a base-10 logarithmic scale, and based on the regression results, the annual percent change (APC) and the respective 95% confidence interval (95%CI) were estimated ^14^.

The percent change is interpreted as follows ^14^:

Increasing trend: positive change and statistically significant model (p-value<0.005).

Decreasing trend: negative change and statistically significant model (p-value<0.005).

Stable trend: non-significant model (p-value>0.005).

In this study, Durbin-Watson values, after correcting for autocorrelation, were reported to ensure reliable trend interpretations-values between 1.5 and 2.5 were considered acceptable. The trend was calculated using Stata version 17.

Individual data

Data analysis was performed using binary logistic regression through the Generalized Linear Model (GLM) method, with a robust covariance matrix estimator. The application of the robust model ensures more accurate estimates and confidence intervals and addresses potential deviations from normality in the residuals. Additionally, GLM significantly enhances the efficiency and robustness of the analysis, allowing for a more precise and flexible interpretation of the data. This approach not only optimizes the modeling process but also reinforces the applicability of logistic regression for large databases with complex structures ^15^ ^,^ ^16^.

For better model fitting, cases with missing data in the dependent variable, as well as those categorized as "unknown" or "not assessed," were excluded. The database was divided into two subsets: the first for cases with grade 1 physical disability and the second for cases with grade 2. Grade 0 physical disability was used as the reference in both analyses. 

The enter method was chosen for variable entry, allowing simultaneous inclusion of all independent variables in the model. For model comparison, the Bayesian Information Criterion (BIC) was evaluated, with lower values indicating better model fit. The software used does not report model fit indicators for pooled regression with imputed data. Therefore, the mean BIC across the five imputed datasets was considered as a comparative measure.

The exponential regression coefficients of the pooled model were interpreted and reported as *odds ratios* (OR), along with their respective 95% confidence intervals (95%CI). Additionally, as a comparative measure to the adjusted OR of the final model, the crude OR were also reported. These analyses were performed in SPSS version 25.

## Results 

The total population was 297,828 cases. After excluding cases classified as "5" (diagnostic error in the discharge type), cases with missing data, cases not classified for physical disability, and cases of transfer in the discharge type variable, the final population included 228,577 leprosy cases analyzed in Brazil.

Table 1 shows that sociodemographic data indicated a predominance of male individuals, especially in the 40-59 age group. Following this trend, the 20-39 age group and those aged 60 or older also accounted for a significant number of cases. Among clinical forms, the dimorphic type was most prevalent, followed by Virchow's and tuberculoid forms. Regarding physical disability at diagnosis, most cases were classified as grade 0, followed by grades 1 and 2.

Table 2 revealed regional differences in the incidence rate of leprosy with grade 2 physical disability per 1 million inhabitants. The North and Central-West regions had the highest rates, while the Southeast and South regions had the lowest. The proportion of new leprosy cases with grade 2 physical disability among individuals aged 0-14 years at diagnosis was highest in the North, followed by the South, Northeast, Southeast, and Central-West.

Table 3 showed a decreasing trend in the proportion of new cases with assessed physical disability at diagnosis in all regions except the North. An increase was observed in new cases with grade 2 physical disability across all regions. The incidence rate decreased only in the Southeast. New cases under 14 years old with grade 2 physical disability increased in Brazil, particularly in the North and Northeast regions.

Table 4 identified factors associated with grade 1 physical disability: individuals aged ≥60 years, adolescents aged 15-19, adults aged 20-39, indeterminate and tuberculoid clinical forms, illiterate and fundamental education levels, and multibacillary forms. 

Table 5 showed factors associated with grade 2 physical disability: male sex, pregnancy, multibacillary form, case entry by transfer, and recurrence. 

**Table 1:** Number of leprosy cases by sociodemographic and clinical variables. Brazil, 2014-2023 (n=228,577)

Characteristics	n (%)
Age group (years)	
0-14	15,913 (5.4)
15-19	12,650 (4.3)
20-39	80,247 (27.4)
40-59	112,528 (38.4)
60+	71,754 (24.5)
Sex	
Male	167,741 (57.2)
Female	125,337 (42.8)
Unknown	14 (0.0)
Pregnancy stage (trimester)	
1st	372 (0.1)
2nd	518 (0.2)
3rd	354 (0.1)
Gestational age unknown	374 (0.1)
No	91,454 (31.2)
Not applicable	193,259 (65.9)
Unknown	6,754 (2.3)
Race/skin color	
White	68,599 (23.7)
Black	36,925 (12.7)
Asian	2,881 (1.0)
Brown	172,973 (59.7)
Indigenous	1,303 (0.4)
Unknown	6,964 (2.4)
Education level	
Illiterate	25,331 (9.4)
Incomplete elementary school (1st-4th grade)	56,974 (21.2)
Complete elementary school (4th grade)	20,873 (7.8)
Incomplete elementary school (5th-8th grade)	42,586 (15.8)
Complete elementary education	17,641 (6.6)
Incomplete high school	18,694 (7.0)
Complete high school	38,018 (14.1)
Incomplete higher education	4,105 (1.5)
Complete higher education	10,016 (3.7)
Unknown	32,975 (12.3)
Not applicable	1,764 (0.7)
Clinical form	
Indeterminate	32,949 (11.6)
Tuberculoid	35,191 (12.4)
Dimorphous	144,225 (51.0)
Virchow’s	53,106 (18.8)
Not classified	17,285 (6.1)
Unknown	185 (0.1)
Physical disability at diagnosis	
Grade 0	154,095 (55.2)
Grade 1	74,657 (26.7)
Grade 2	26,594 (9.5)
Not assessed	23,892 (8.6)
Operational classification	
Paucibacilary	64,187 (21.9)
Multibacillary	228,518 (78.1)
Unknown	34 (0.0)
Input mode	
New cases	233,132 (79.6)
Transfer from the same municipality (other center)	6,365 (2.2)
Transfer from another municipality (same state)	9,421 (3.2)
Transfer from another state	5,471 (1.9)
Transfer from another country	166 (0.1)
Recurrence	13,473 (4.6)
Other re-entries	24,307 (8.3)
Unknown	404 (0.1)
Mode of detection of new cases	
Referral	107,949 (45.6)
Spontaneous demand	93,154 (39.3)
Community examination	9,386 (4.0)
Contacts examination	20,396 (8.6)
Other modes	5,191 (2.2)
Unknown	818 (0.3)
Bacilloscopy	
Positive	55,369 (27.2)
Not performed	67,281 (33.0)
Negative	74,700 (36.7)
Unknown	6,408 (3.1)
Initial treatment regimen	
Polychemotherapy/paucibacilary/6 doses	63,256 (21.6)
Polychemotherapy/multibacillary/12 doses	223,065 (76.3)
Other alternative regimens	5,931 (2.1)
Reactional episode during treatment	
Type 1	27,210 (12.5)
Type 2	8,887 (4.1)
Type 1 and 2	3,964 (1.8)
No information	177,254 (81.6)

**Table 2:** Operational Indicators of leprosy associated with the degree of disability. Brazil, 2014-2023 (n=228,577)

Indicator	2014	2015	2016	2017	2018	2019	2020	2021	2022	2023^a^	Total
Proportion of new leprosy cases with disability grade assessed at diagnosis											
North	92.8	92.3	92.8	94.1	94.5	93.8	92.9	94.3	92.4	93.0	93.3
Northeast	84.9	85.3	84.9	84.0	83.9	83.3	82.9	83.6	81.7	83.7	84.0
Southeast	94.4	93.6	93.3	92.2	92.8	91.0	91.3	90.3	88.2	84.0	91.9
South	94.6	95.1	95.1	94.9	93.8	93.7	87.6	91.2	88.2	83.4	92.9
Central-West	88.4	88.6	88.2	89.8	86.9	88.0	83.6	84.5	83.9	82.0	87.1
Brazil	88.8	88.7	88.6	88.6	88.1	87.7	86.2	87.0	85.1	85.0	87.8
Proportion of new leprosy cases with grade 2 physical disability at diagnosis											
North	6.7	6.7	7.4	8.4	8.7	9.4	8.8	12.3	11.5	12.0	8.6
Northeast	6.8	7.0	6.9	7.8	8.1	8.5	8.7	9.9	9.6	9.9	8.0
Southeast	11.2	10.6	13.5	11.9	11.6	15.0	14.9	14.5	16.1	17.9	13.1
South	10.4	10.1	11.8	13.3	14.4	15.0	12.2	13.9	15.1	13.7	12.7
Central-West	6.8	6.3	6.6	6.0	6.0	8.8	9.7	9.7	11.0	11.5	7.6
Brazil	7.6	7.4	8.1	8.3	8.4	9.8	10.0	11.2	11.5	12.0	9.0
Incidence rate of leprosy with grade 2 disability at diagnosis per 1,000,000 inhabitants											
North	22.13	18.79	19.39	22.64	26.23	25.07	14.30	20.36	17.87	5.53	19.12
Northeast	14.31	13.81	11.54	13.80	14.18	14.51	9.60	11.53	12.03	3.09	11.81
Southeast	5.70	4.77	5.48	4.76	4.50	5.74	3.98	4.31	4.91	1.40	4.54
South	3.54	3.37	3.17	3.39	3.60	3.80	2.05	2.70	2.81	0.71	2.90
Central-West	24.45	20.46	17.75	18.34	21.70	30.86	19.21	16.88	20.64	6.78	19.61
Brazil	10.57	9.45	8.95	9.63	10.23	11.49	7.32	8.44	8.92	2.55	8.72
Proportion of new leprosy cases in the 0-14 years population with grade 2 physical disability at diagnosis											
North	2.9	2.8	3.8	3.4	3.1	4.4	5.1	5.9	6.3	3.8	3.7
Northeast	2.4	2.6	1.6	3.1	2.8	3.6	4.1	3.7	5.6	6.2	3.0
Southeast	1.1	6.1	1.9	3.0	-	1.5	3.8	2.3	10.7	4.5	3.0
South	-	-	16.7	10.5	10.0	-	-	-	-	-	3.5
Central-West	2.0	1.9	1.9	2.9	2.7	4.1	7.4	3.2	3.1	-	2.8
Brazil	2.3	2.8	2.3	3.2	2.6	3.6	4.7	4.0	5.9	4.0	3.2

a Data relating to the months of January to May.

**Table 3:** Annual percent change (APC) and confidence intervals (95%CI) of leprosy operational indicators associated with disability grade. Brazil, 2014-2023 (n=228,577)

Variables	APC (95%CI)	p-value	D-W^a^	Interpretation
Proportion of new leprosy cases with disability grade assessed at diagnosis				
North	0.0 (-0.2; 0.3)	0.711	1.921	Stable
Northeast	-0.4 (-0.5; -0.3)	<0.001	1.925	Decrease
Southeast	-1.2 (-1.8; -0.5)	0.003	1.184	Decrease
South	-1.3 (-1.8; -0.7)	<0.001	1.619	Decrease
Central-West	-0.9 (-1.3; -0.5)	<0.001	1.723	Decrease
Brazil	-0.5 (-0.7; -0.4)	<0.001	1.763	Decrease
Proportion of new leprosy cases with grade 2 physical disability at diagnosis				
North	7.5 (6.3; 8.8)	<0.001	2.448	Increase
Northeast	4.9 (4.1; 5.7)	<0.001	2.066	Increase
Southeast	5.2 (3.7; 6.8)	<0.001	2.442	Increase
South	3.5 (0.4; 6.7)	0.032	1.680	Increase
Central-West	7.3 (2.9; 11.8)	0.005	1.615	Increase
Brazil	6.0 (5.1; 6.9)	<0.001	1.679	Increase
Incidence rate of leprosy with grade 2 disability at diagnosis per 1,000,000 inhabitants				
North	-1.8 (-7.2; 3.9)	0.467	2.007	Stable
Northeast	-2.6 (-6.0; 0.9)	0.121	2.120	Stable
Southeast	-2.4 (-4.3; -0.3)	0.028	2.791	Decrease
South	-3.9 (-8.3; 0.6)	0.080	2.104	Stable
Central-West	-1.0 (-7.0; 5.4)	0.713	1.856	Stable
Brazil	-2.0 (-5.4; 1.5)	0.210	2.098	Stable
Proportion of new leprosy cases in the 0-14 years population with grade 2 physical disability at diagnosis				
North	7.9 (3.1; 13.0)	0.005	1.680	Increase
Northeast	12.5 (8.4; 16.7)	<0.001	2.064	Increase
Southeast	10.0 (-1.6; 23.0)	0.083	1.519	Stable
South^b^	-	-	-	-
Central-West	11.0 (-0.6; 24.0)	0.061	1.797	Stable
Brazil	9.9 (7.5; 12.5)	<0.001	1.933	Increase

a D-W: Durbin Watson; ^b^ Insufficient data for analysis.

**Table 4:** Crude and adjusted odds ratio (OR) with 95% confidence intervals (95%CI) for Grade 1 leprosy disability by sociodemographic and clinical variables. Brazil, 2014-2023 (n=228,577)

Predictors	Crude OR (95%CI)	p-value	Adjusted OR (95%CI)	p-value
Age group (years)				
60+	4.17 (3.96; 4.38)	<0.001	3.36 (3.15; 3.58)	<0.001
15-19	1.64 (1.53; 1.75)	<0.001	1.64 (1.51; 1.79)	<0.001
20-39	2.37 (2.25; 2.49)	<0.001	2.04 (1.91; 2.18)	<0.001
40-59	3.30 (3.14; 3.47)	<0.001	2.62 (2.47; 2.79)	<0.001
0-14	1.00		1.00	
Sex				
Male	1.32 (1.30; 1.34)	<0.001	0.97 (0.64; 1.49)	0.879
Female	1.00		1.00	
Pregnant person				
Yes	1.07 (0.61; 1.89)	0.747	1.32 (1.13; 1.54)	0.001
No	1.00		1.00	
Race/skin color				
Black	1.0 (0.97; 1.03)	0.787	1.03 (0.99; 1.08)	0.098
Brown	0.89 (0.87; 0.91)	<0.001	0.95 (0.93; 0.98)	0.001
Asian	1.05 (0.96; 1.14)	0.265	0.99 (0.89; 1.11)	0.907
Indigenous	1.01 (0.88; 1.15)	0.908	1.17 (0.99; 1.38)	0.070
White	1.00		1.00	
Education level				
Illiterate	2.00 (1.91; 2.10)	<0.001	1.50 (1.41; 1.61)	<0.001
Elementary school	1.44 (1.38; 1.51)	<0.001	1.36 (1.29; 1.44)	<0.001
High school	1.11 (1.06; 1.16)	<0.001	1.15 (1.09; 1.22)	<0.001
Higher education	1.00		1.00	
Clinical form				
Indeterminate	0.37 (0.35; 0.39)	<0.001	0.82 (0.76; 0.88)	<0.001
Tuberculoid	0.44 (0.42; 0.46)	<0.001	0.95 (0.89; 1.02)	0.131
Dimorphous	1.46 (1.40; 1.52)	<0.001	1.11 (1.06; 1.17)	<0.001
Virchow’s	1.94 (1.86; 2.03)	<0.001	1.43 (1.35; 1.52)	<0.001
Not classified	1.00		1.00	
Operational classification				
Multibacillary	4.55 (4.43; 4.67)	<0.001	2.05 (1.94; 2.16)	<0.001
Paucibacilary	1.00		1.00	
Input mode				
Referral	1.80 (1.75; 1.85)	<0.001	1.39 (1.28; 1.51)	<0.001
Recurrence	1.52 (1.48; 1.58)	<0.001	1.52 (1.36; 1.69)	<0.001
New cases	1.00		1.00	
Mode of detection of new cases				
Referral	0.80 (0.75; 0.86)	<0.001	0.95 (0.88; 1.02)	0.171
Spontaneous demand	0.79 (0.74; 0.85)	<0.001	0.91 (0.84; 0.99)	0.020
Community examination	0.92 (0.85; 1.00)	0.059	1.16 (1.06; 1.27)	0.002
Contacts examination	0.79 (0.73; 0.85)	<0.001	0.87 (0.80; 0.95)	0.002
Other modes	1.00		1.00	
Bacilloscopy				
Positive	1.56 (1.53; 1.60)	<0.001	1.09 (1.05; 1.12)	<0.001
Not performed	1.05 (1.03; 1.08)	<0.001	0.91 (0.88; 0.94)	<0.001
Negative	1.00		1.00	
Number of affected nerves	1.49 (1.48; 1.50)	<0.001	1.41 (1.40; 1.42)	<0.001

**Table 5:** Crude and adjusted odds ratios (OR) with 95% confidence intervals (CI) for grade 2 physical disability in leprosy by sociodemographic and clinical variables. Brazil, 2014-2023 (n=228,577)

Predictors	Crude OR (95%CI)	p-value	Adjusted OR (95%CI)	p-value
Age group (years)				
60+	7.04 (6.43; 7.72)	<0.001	4.10 (3.65; 4.59)	<0.001
15-19	1.95 (1.74; 2.20)	<0.001	1.85 (1.61; 2.13)	<0.001
20-39	2.90 (2.65; 3.18)	<0.001	2.08 (1.86; 2.33)	<0.001
40-59	4.17 (3.80; 4.56)	<0.001	2.58 (2.31; 2.89)	<0.001
0-14	1.00		1.00	
Sex				
Male	2.19 (2.13; 2.25)	<0.001	1.56 (1.51; 1.62)	<0.001
Female	1.00		1.00	
Pregnant person				
Yes	0.54 (0.43; 0.69)	<0.001	1.11 (0.86; 1.42)	0.420
No	1.00		1.00	
Race/skin color				
Black	1.05 (1.00; 1.10)	0.032	1.02 (0.96; 1.07)	0.595
Brown	0.86 (0.83; 0.89)	<0.001	0.93 (0.89; 0.96)	<0.001
Asian	0.85 (0.74; 0.98)	0.025	0.79 (0.66; 0.94)	0.007
Indigenous	1.06 (0.88; 1.29)	0.516	1.20 (0.96; 1.49)	0.117
White	1.00		1.00	
Education level				
Illiterate	4.21 (3.84; 4.63)	<0.001	2.80 (2.50; 3.13)	<0.001
Elementary school	2.35 (2.17; 2.53)	<0.001	2.07 (1.88; 2.27)	<0.001
High school	1.39 (1.27; 1.51)	<0.001	1.44 (1.30; 1.59)	<0.001
Higher education	1.00		1.00	
Clinical form				
Indeterminate	0.12 (0.11; 0.14)	<0.001	0.39 (0.34; 0.44)	<0.001
Tuberculoid	0.23 (0.21; 0.25)	<0.001	0.71 (0.63; 0.79)	<0.001
Dimorphous	1.10 (1.04; 1.17)	0.001	0.75 (0.70; 0.81)	<0.001
Virchow’s	2.42 (2.27; 2.57)	<0.001	1.43 (1.04; 1.33)	<0.001
Not classified	1.00		1.00	
Operational classification				
Multibacillary	11.41 (11.05; 11.78)	<0.001	1.99 (1.54; 2.59)	<0.001
Paucibacilary	1.00		1.00	
Input mode				
Referral	2.79 (2.69; 2.88)	<0.001	1.77 (1.68; 1.86)	<0.001
Recurrence	1.70 (1.62; 1.78)	<0.001	1.34 (1.26; 1.42)	<0.001
New cases	1.00		1.00	
Mode of detection of new cases				
Referral	0.45 (0.42; 0.49)	<0.001	0.85 (0.74; 0.96)	0.016
Spontaneous demand	0.35 (0.32; 0.38)	<0.001	0.60 (0.52; 0.70)	<0.001
Community examination	0.35 (0.31; 0.39)	<0.001	0.70 (0.58; 0.85)	0.002
Contacts examination	0.23 (0.21; 0.26)	<0.001	0.41 (0.35; 0.49)	<0.001
Other modes	1.00		1.00	
Bacilloscopy				
Positive	1.85 (1.76; 1.94)	<0.001	0.92 (0.86; 0.99)	0.024
Not performed	0.92 (0.88; 0.96)	<0.001	0.77 (0.73; 0.81)	<0.001
Negative	1.00		1.00	
Initial treatment regimen				
Polychemotherapy/paucibacilary/6 doses	0.05 (0.04; 0.05)	<0.001	0.56 (0.43; 0.73)	<0.001
Polychemotherapy/multibacillary/12 doses	0.54 (0.50; 0.58)	<0.001	0.86 (0.77; 0.96)	0.007
Other alternative regimens	1.00		1.00	
Number of affected nerves	1.61 (1.59; 1.62)	<0.001	1.53 (1.52; 1.55)	<0.001

## Discussion 

The epidemiological data assessment identified a total of 228,577 diagnosed cases of leprosy across the regions of Brazil during the analyzed period. The results indicated lower odds of presenting grade 1 or grade 2 physical disabilities associated with female sex, tuberculoid clinical form, residence in the Southeast and South regions, and higher education level. Conversely, new leprosy cases in individuals aged 40-59 years, with lower education levels, and multibacillary clinical form had higher odds of developing grade 1 or 2 physical disabilities at the time of diagnosis.

The study has limitations related to missing data, especially regarding the assessment of disability at diagnosis. The absence of this information prevents a more complete analysis and may introduce bias if data loss is not random. To address this issue, a multiple imputation procedure was adopted, assuming that the losses were missing at random (MAR), a common approach in studies of this nature. Additionally, the quality of the data, derived from routine surveillance activities, does not always ensure consistent collection of all relevant information, such as health service characteristics and patient perceptions, limiting the depth of analysis. Despite these limitations, multiple imputation helped minimize the impact of missing data, although improvements in data collection and analysis are needed in future studies.

This study stands out by filling a gap in the literature by exploring the relationship between leprosy physical disability grades and associated factors using a comprehensive, nationally representative approach. Recent data were analyzed, allowing the investigation of the spatial and temporal distribution of the disease and the identification of geographic patterns influenced by social and environmental determinants. 

A predominance of cases was observed among males in comparison to female individuals. The data suggest that men have higher exposure to *Mycobacterium leprae*, possibly due to lower delivery of health care, influenced by cultural factors. This neglect may result in delayed diagnosis, increasing the risk of developing physical disabilities. Studies show that male populations face programmatic barriers in health services, which increases their vulnerability to late diagnosis and leprosy-related complications ^17^ ^,^ ^18^.

In this study, the South and Southeast regions presented the highest proportions of new cases with grade 2 physical disability. This proportion is considered high when compared to the Ministry of Health parameters: <5.0% as low, 5.0%-9.9% as medium, and >10.0% as high ^8^. It is not possible to precisely justify the reasons for this scenario in these regions; however, a higher concentration of grade 2 disability among individuals under 15 years of age may help explain this pattern. 

In recent years, several milestones have emerged aiming to reduce cases in this population, such as: the Global Leprosy Strategy (2016-2020); the implementation of surveillance for grade 2 physical disability in individuals under 15 in Brazil in 2018; the Global Leprosy Strategy (2021-2030); and the implementation of Technical Note N1/2023, which recommends and guides the implementation of grade 2 surveillance for all new cases ^19^. 

Thus, this high proportion is concerning, indicating the need for adjustments in strategies related to early diagnosis and treatment to minimize the impact of disabilities associated with leprosy ^20^. It is important to enhance simplified neurological assessment and use it primarily as a tool to support care for people affected by leprosy, both during and after multidrug therapy. It is also necessary to strengthen active case-finding actions to identify new cases early, investigate close contacts, and implement additional preventive measures, such as chemoprophylaxis, aiming to reduce transmission and prevent the occurrence of new disabling cases.

The lowest odds of grade 1 and 2 physical disabilities were observed in cases residing in the Southeast and South regions. Data indicated higher proportions of these disabilities in municipalities with high leprosy incidence rates, predominantly located in the North and Northeast regions. These findings are consistent with studies highlighting the relationship between high disease burden and a higher prevalence of disabling forms in areas with limited access to early diagnosis and appropriate treatment ^21^ ^,^ ^22^ ^,^ ^23^. 

The Southeast and South regions of Brazil stand out for having higher socioeconomic indicators, which provide a more robust infrastructure in terms of health and essential services ^24^. This contrasts with the North, Northeast, and Central-West regions, which have historically faced significant socioeconomic challenges, such as a lack of resources and access to quality health care services. Therefore, the lowest leprosy prevalence observed in the South region partly reflects the higher level of development in these areas ^25^ ^,^ ^26^. Broader access to information, prevention, and appropriate treatment helps reduce cases, whereas regions with lower development indices face challenges that exacerbate the disease situation, including later diagnoses and higher prevalence of disabling forms.

This study indicates that higher levels of education are less associated with the presence of physical disabilities at diagnosis, confirming existing studies ^27^ ^,^ ^28^. As noted by Soares et al. ^29^, lower education levels are associated with greater vulnerability and neglect, maintaining leprosy as a problem that accompanies different life cycles. Higher educational attainment is associated with improved understanding of health conditions and, consequently, better access to and use of health services. Periodic assessments and self-care practices are factors that can help prevent the worsening of clinical symptoms.

Higher odds of leprosy-related disabilities were observed in individuals over 40 years old. This age group, often active in the workforce, may face occupational limitations due to disabilities caused by the disease. Such restrictions can result in work absences, early retirements, and a significant reduction in quality of life, affecting both workers and their families, as reported by Ramos et al. ^30^. In this context, implementing measures aimed at reducing physical sequelae and reintegrating these individuals into the workforce would be transformative, as occupational reintegration allows people affected by leprosy to resume professional activities, thereby reducing income loss and social exclusion.

Leprosy in multibacillary form is associated with an increased risk of developing grade 1 or 2 Physical Disabilities at the time of diagnosis. Studies conducted in Brazilian municipalities show that the operational classification of the disease has a statistically significant relationship with the occurrence of these disabilities, presenting a 99.0% higher risk of developing sequelae compared to paucibacillary. Ongoing transmission in areas with limited access to health care contributes to a higher number of cases, which carry an elevated risk of evolving into disabling forms. This underscores the importance of early diagnosis, as well as the implementation of specific strategies to identify and treat these cases, aiming to reduce the risk of physical disabilities and improve patient outcomes ^31^ ^,^ ^17^.

In conclusion, the findings reveal that even though leprosy is one of the oldest diseases ever reported, advances in diagnosis and treatment remain critically important, along with health education and inclusive public policies applied consistently, which continue to be necessary to reduce cases in regions of higher endemicity. The geographic and temporal distribution of leprosy reflects inequalities in access to diagnosis and treatment, especially in economically vulnerable regions. The challenge is not only to increase early detection but also to ensure that health care reaches everyone equitably, and that society understands the importance of prevention, continuous treatment, and patient integration into the community.

## Data Availability

The database and variable descriptions are available at https://doi.org/10.48331/scielodata.UOZXWF.
