# Supplementary material for: Temporal trend and factors associated with physical disability due to leprosyl: a time series study, Brazil, 2014-2023
Source: Epidemiol Serv Saude. 2026 May 1;35:e20240899. doi: 10.1590/S2237-96222026v35e20240899.en (PMC13138604; doi:10.1590/S2237-96222026v35e20240899.en)
Supplement: Translation

# Translation: Tendência temporal e fatores associados à incapacidade física da hanseníase: estudo de série temporal, Brasil, 2014-2023

**DOI:** 10.1590/S2237-96222026v35e20240899.pt

**Authors:** Everton dos Santos Araújo, Jefferson Felipe Calazans Batista, Yasmim Ingrid Nascimento Santos, Marcos Antonio Almeida-Santos

**Affiliations:** ¹ Universidade Tiradentes, Programa de Pós-Graduação em Saúde e Meio Ambiente, Aracaju, SE, Brasil

**Keywords:** Hanseníase Multibacilar, Hanseníase Tuberculoide, Mycobacterium leprae, Estudos de Séries Temporais, Estudos Transversais.

## Abstract

**Objetivo:**

Analisar a tendência temporal e os fatores associados à incapacidade física da hanseníase no Brasil no período de 2014 a 2023.

**Métodos:**

Estudo de série temporal subdividido em análise dos indicadores operacionais e dos fatores associados ao grau de incapacidade da hanseníase no Brasil. Os dados foram extraídos do Sistema de Informação de Agravos de Notificação. Foi calculada a taxa de incidência de hanseníase com incapacidade física grau 2 por 1 milhão de habitantes. Os fatores associados foram medidos pela regressão logística, que gera a razão de chances (*odds ratio*, OR), a tendência pela regressão de Prais-Winsten e a variação percentual anual.

**Resultados:**

Foram analisados 228.577 casos de hanseníase no Brasil entre 2014 e 2023. As regiões Centro-Oeste e Norte apresentaram taxa de 19,61/1 milhão e 19,12/1 milhão, respectivamente. A variação percentual anual foi de 6,0 (intervalo de confiança de 95% - IC95% 5,1; 6,9) para os casos novos com grau 2 de incapacidade no Brasil. Os fatores associados ao grau de incapacidade 1 foram: ≥60 anos (OR 3,36; IC95% 1,03; 3,15), forma tuberculoide (OR 0,95; IC95% 0,89; 1,04) e baixa escolaridade (OR 1,50; IC95% 1,03; 1,41). Para o grau 2, os fatores associados foram sexo masculino (OR 1,56; IC95% 1,02; 1,51), gestação (OR 1,11; IC95% 0,86; 1,13) e forma multibacilar (OR 1,99; IC95% 1,14; 1,54).

**Conclusão:**

Em suma, menores chances de apresentar incapacidades físicas de grau 1 ou grau 2 associadas ao sexo feminino, à forma clínica tuberculoide, à residência nas regiões Sudeste e Sul e à condição de nível superior.

Ethical aspects

Esta pesquisa utilizou bancos de dados de domínio público e anonimizados.

## Introdução 

O *Mycobacterium leprae* é o causador da hanseníase, doença que representa um grande desafio para à saúde pública do Brasil. Considerada uma das principais endemias do país, a hanseníase interfere na qualidade de vida dos indivíduos afetados e impõe custos socioeconômicos elevados. O grau de incapacidade física, refletido pela presença de limitações físicas nos pacientes, é um indicador crucial para mensurar o impacto da doença e a eficácia das intervenções de saúde pública. Entre 2014 e 2023, o Brasil testemunhou variações nos índices de incapacidades associadas à hanseníase, o que demanda uma análise aprofundada das tendências temporais e dos fatores que influenciam esses resultados ^1^.

O Brasil é o segundo país com maior carga de casos novos. Essa situação pode estar associada a fatores diversos, como o acesso inadequado a serviços de saúde, a falta de diagnóstico precoce e as desigualdades socioeconômicas, contextos que limitam a prevenção e o tratamento eficaz ^2^. Monitorar indicadores operacionais é essencial para orientar políticas públicas e melhorar a qualidade de vida dos pacientes, enquanto o fortalecimento das redes de apoio e a colaboração entre setores são fundamentais para reduzir incapacidades e promover a dignidade dos afetados ^3^ ^,^ ^4^. 

A desigualdade regional é um fator determinante para a hanseníase no Brasil, as regiões Norte e Nordeste, por exemplo, concentram altas taxas de incidência e de incapacidades físicas. Esses dados indicam uma correlação entre a pobreza, a baixa escolaridade e a maior vulnerabilidade à hanseníase, apesar do investimento em estratégias de vigilância epidemiológica e em campanhas de conscientização. No entanto, a efetividade dessas ações ainda é questionável, considerando os dados sobre a persistência das incapacidades físicas. A desarticulação entre as políticas de saúde e os serviços de atenção básica pode ser um entrave à implementação efetiva de estratégias que visem à redução das incapacidades ^5^ ^,^ ^6^.

Ademais, a atuação do sistema de saúde relacionado à incapacidade física da hanseníase no Brasil nos últimos dez anos revela desafios complexos e interconectados. Assim sendo, objetivamos analisar a tendência temporal e os fatores associados à incapacidade física da hanseníase no Brasil no período de 2014 a 2023.

## Métodos 

Delineamento do estudo

Estudo de série temporal exploratório e analítico, com abordagem quantitativa, subdividido em análise temporal dos indicadores operacionais e dos fatores associados ao grau de incapacidade da hanseníase no Brasil ^7^.

Contexto

Foram utilizados dados públicos referentes aos casos de hanseníase no Brasil e suas macrorregiões no período de janeiro de 2014 a maio de 2023.

Participantes

Foram considerados os casos confirmados de hanseníase na população brasileira. Foram excluídos os casos classificados como "erro de diagnóstico" na variável "tipo de saída", por não serem caracterizados como caso de hanseníase.

Variáveis

Foram selecionadas as seguintes variáveis:

Sociodemográficas

Idade detalhada (em anos);

Sexo (masculino; feminino);

Macrorregião do país (Norte; Nordeste; Sudeste; Sul; Centro-Oeste);

Ano de diagnóstico (2014-2023);

Raça/cor da pele (branca; preta; parda; amarela; indígena);

Escolaridade (analfabeto; ensino fundamental incompleto; ensino fundamental completo; ensino médio incompleto; ensino médio completo; ensino superior incompleto; ensino superior completo); e

Gestante (1º trimestre; 2º trimestre; 3º trimestre; idade gestacional ignorada; não; não se aplica; ignorado).

Clínicas:

Forma clínica (indeterminada; tuberculoide; dimorfa; virchowiana; não classificado; ignorado);

Grau de incapacidade no momento do diagnóstico (grau 0; grau 1; grau 2; não avaliado; ignorado);

Classificação operacional (caso novo; transferência do mesmo município [outra unidade federativa]; transferência de outro município [mesma unidade federativa]; transferência de outro estado; transferência de outro país; recidiva; outros reingressos; ignorado);

Modo de detecção de caso novo (encaminhamento; demanda espontânea; exame de coletividade; exame de contatos; outros modos; ignorado);

Baciloscopia (positiva; negativa; não realizada; não se aplica);

Esquema terapêutico inicial (PQT/PB/6 doses; PQT/MB/12 doses; outros esquemas substitutos);

Episódio reacional durante o tratamento (reação tipo 1; reação tipo 2; reação tipo 1 e 2);

Número de lesões cutâneas;

Número de contatos registrados;

Número de contatos examinados;

Número de doses supervisionadas; e

Número de nervos afetados.

A variável grau de incapacidade no momento do diagnóstico foi considerada como dependente. Essa variável foi expressa como "incapacidade física". Nos dados agregados, foram calculados alguns indicadores operacionais da hanseníase associadas ao grau de incapacidade, seguindo os padrões do Ministério da Saúde, a saber ^8^:

Proporção de casos novos de hanseníase com grau de incapacidade física avaliado no momento do diagnóstico.

Proporção de casos novos de hanseníase com incapacidade física grau 2 no momento do diagnóstico.

Taxa de incidência de hanseníase com incapacidade física grau 2 no momento do diagnóstico por 1 milhão de habitantes.

Proporção de casos novos de hanseníase, na população de 0-14, com incapacidade física grau 2 no momento do diagnóstico.

Fonte de dados/mensuração

Os dados de hanseníase foram extraídos do Departamento de Informática do Sistema Único de Saúde por meio do Sistema de Informação de Agravos de Notificação ^9^, Doenças e Agravos de Notificação, enquanto a população residente para o cálculo do indicador 3 foi proveniente da projeção da população de 2010-2060 do Instituto Brasileiro de Geografia e Estatística ^10^. Todos os dados foram acessados em julho de 2024 da seguinte forma: 

Acesso aos bancos anuais (2014-2023) das fichas de notificação no site do Departamento de Informática do Sistema Único de Saúde - dados em formato *.dbc.*

Importação dos bancos baixados para o TabWin, para conversão de *.dbc* para *.dbf.*

União dos bancos de dados anuais em um banco único.

Tabulação dos dados para os indicadores operacionais, segundo padrão do Ministério da Saúde.

Exportação dos microdados para tratamento em planilha do Microsoft Excel.

O banco de dados da pesquisa está disponível online ^11^. 

Detalhamento das variáveis

As seguintes variáveis independentes sofreram modificações: idade detalhada → faixa etária (0 a 14 anos; 15-19 anos; 20-39 anos; 40-59 anos; ≥60 anos); escolaridade → aglutinada em analfabeto, ensino fundamental, médio e superior; modo de entrada → todas as categorias de transferência aglutinadas em um único grupo, "transferência"; gestante → primeiro a terceiro trimestre + idade ignorada aglutinado em "sim". 

Na variável "forma clínica", existiam 39 indivíduos cujo grupo foi inserido como "6", valor não condizente com o que é apresentado na ficha de notificação e no dicionário de dados. Portanto, essa classificação errada foi transformada em dado branco. Para a análise dos dados, as variáveis com agrupamentos "ignorados" ou "não se aplica" foram também transformadas em dados brancos.

Viés

Considerando a presença de dados faltantes no estudo e visando à melhoria do modelo preditivo, foi realizado um procedimento de imputação múltipla partindo do pressuposto de que as perdas são do tipo ao acaso (do inglês, *missing at random*), fato comum em estudos dessa natureza ^12^. Portanto, por meio da inserção de todas as variáveis disponíveis e usando o método de correspondência preditiva (do inglês, *predictive mean matching*) foram propostos cinco modelos de imputação ^13^. 

Análise dos dados

*Dados agregados*

A tendência temporal foi estimada pela regressão de Prais-Winsten. Todos os indicadores foram transformados em escala logarítmica de base dez e, ancorando-se nos resultados da regressão, estimaram-se a variação percentual anual e o respectivo intervalo de confiança de 95% (IC95%) ^14^.

A variação percentual é interpretada da seguinte forma ^14^:

Tendência de crescimento: variação positiva e modelo estatisticamente significativo (p-valor<0,005).

Tendência de redução: variação negativa e modelo estatisticamente significativo (p-valor<0,005).

Tendência estacionária: modelo não significativo (p-valor>0,005).

Nesta pesquisa, os valores de Durbin-Watson, após a correção da autocorrelação, foram reportados, a fim de assegurar as interpretações da tendência - consideraram-se como confiáveis valores entre 1,5 e 2,5. A tendência foi calculada no Stata versão 17.

Dados individualizados

A análise dos dados foi realizada utilizando regressão logística binária pelo método Generalized Linear Model (GLM), com estimador robusto de matriz de covariância. A aplicação do modelo robusto assegura estimativas e intervalos de confiança mais precisos, além de contornar possíveis desvios de normalidade dos resíduos. Além disso, os GLM aprimoram significativamente a eficiência e a robustez da análise, o que permite uma interpretação mais precisa e flexível dos dados. Tal abordagem não só otimiza o processo de modelagem, mas também reforça a aplicabilidade da regressão logística para grandes bancos de dados com estruturas complexas ^15^ ^,^ ^16^.

Para melhor ajuste dos modelos, foram excluídos os casos com dados em brancos na variável dependente, além dos categorizados como "ignorado" ou "não avaliado". O banco de dados foi dividido em dois, sendo o primeiro para os casos incapacidade física grau 1 e o segundo somente para o grau 2. Foi considerado com referência em ambas as análises a incapacidade física de grau 0. 

O *enter* foi escolhido como método de entrada, permitindo a inclusão simultânea de todas as variáveis independentes no modelo. Para a comparação dos modelos, foram avaliados o Bayesian Information Criterion, no qual menores valores indicam melhor ajuste do modelo. O programa usado para análise dos dados não informa, na regressão com dados imputados, os indicadores de ajustes no modelo agrupado (*pooled*). Portanto, considerou-se a média do critério bayesiano dos cinco bancos imputados como medida comparativa.

Os coeficientes da regressão exponenciais do modelo agrupado foram interpretados e reportados como razão de chances (*odds ratio*, OR) e IC95%. Além disso, como medida comparativa às OR ajustadas do modelo final, foram reportadas também as OR brutas. Essas análises foram realizadas no SPSS versão 25.

## Resultados 

A população total foi 297.828. Após a exclusão de casos classificados como "5" (erro de diagnóstico no tipo de saída), de casos em branco, de casos não classificados na variável incapacidade física e da transferência da variável tipo de saída, a população final foi de 228.577 casos analisados de hanseníase no Brasil.

Na Tabela 1, os dados sociodemográficos apontaram predominância entre indivíduos do sexo masculino, especialmente na faixa etária de 40-59 anos. Seguindo essa tendência, as faixas de 20-39 anos e de 60 anos ou mais também concentraram uma quantidade significativa de casos. Entre as formas clínicas, a dimorfa foi prevalente, seguida pela virchowiana e pela tuberculoide. Em relação à incapacidade física no diagnóstico, a maioria dos casos foi classificada como grau 0, seguida pelos graus 1 e 2.

A Tabela 2 revelou diferenças regionais na taxa de incidência de hanseníase com incapacidade física grau 2 por 1 milhão de habitantes. Nas regiões Norte e Centro-Oeste, foram registradas maiores taxas, enquanto as regiões Sudeste e Sul apresentaram as menores. A proporção de casos novos de hanseníase com incapacidade física grau 2 na população de 0-14 anos no momento do diagnóstico foi maior na região Norte, seguida pelas regiões Sul, Nordeste, Sudeste e Centro-Oeste.

Na Tabela 3, observou-se tendência de diminuição da proporção de casos novos com incapacidade física avaliada no diagnóstico em todas as regiões, exceto na região Norte. Foi observado crescimento do indicador de casos novos com incapacidade física grau 2 em todas as localidades. A taxa de incidência apresentou tendência de diminuição somente na região Sudeste. Os casos novos em menores de 14 anos com incapacidade física grau 2 apresentaram crescimento no Brasil, nas regiões Norte e Nordeste.

Na Tabela 4, os fatores associados à incapacidade física grau 1 foram: indivíduos com 60 anos ou mais; jovens de 15-19 anos; adultos de 20-39 anos; forma indeterminada e tuberculoide; escolaridade analfabeta e fundamental; e forma multibacilar. 

Na Tabela 5, os fatores associados à incapacidade física grau 2 foram: sexo masculino; gestantes; forma multibacilar, ingresso por transferência e recidiva. 

**Tabela 1:** Número de casos de hanseníase segundo variáveis sociodemográficas e clínicas. Brasil, 2014-2023 (n=228.577)

Características	n (%)
Faixa etária (anos)	
0-14	15.913 (5,4)
15-19	12.650 (4,3)
20-39	80.247 (27,4)
40-59	112.528 (38,4)
60+	71.754 (24,5)
Sexo	
Masculino	167.741 (57,2)
Feminino	125.337 (42,8)
Ignorado	14 (0,0)
Período de gestação (trimestre)	
1º	372 (0,1)
2º	518 (0,2)
3º	354 (0,1)
Idade gestacional ignorada	374 (0,1)
Não	91.454 (31,2)
Não se aplica	193.259 (65,9)
Ignorado	6.754 (2,3)
Raça/cor da pele	
Branca	68.599 (23,7)
Preta	36.925 (12,7)
Amarela	2.881 (1,0)
Parda	172.973 (59,7)
Indígena	1.303 (0,4)
Ignorada	6.964 (2,4)
Escolaridade	
Analfabeto	25.331 (9,4)
1ª-4ª série incompleta do ensino fundamental	56.974 (21,2)
4ª série completa do ensino fundamental	20.873 (7,8)
5ª-8ª série incompleta do ensino fundamental	42.586 (15,8)
Ensino fundamental completo	17.641 (6,6)
Ensino médio incompleto	18.694 (7,0)
Ensino médio completo	38.018 (14,1)
Educação superior incompleta	4.105 (1,5)
Educação superior completa	10.016 (3,7)
Ignorado	32.975 (12,3)
Não se aplica	1.764 (0,7)
Forma clínica	
Indeterminada	32.949 (11,6)
Tuberculoide	35.191 (12,4)
Dimorfa	144.225 (51,0)
Virchowiana	53.106 (18,8)
Não classificado	17.285 (6,1)
Ignorado	185 (0,1)
Incapacidade física no diagnóstico	
Grau 0	154.095 (55,2)
Grau 1	74.657 (26,7)
Grau 2	26.594 (9,5)
Não avaliado	23.892 (8,6)
Classificação operacional	
Paucibacilar	64.187 (21,9)
Multibacilar	228.518 (78,1)
Ignorado	34 (0,0)
Modo de entrada	
Caso novo	233.132 (79,6)
Transferência do mesmo município (outra unidade)	6.365 (2,2)
Transferência de outro município (mesma Unidade da Federação)	9.421 (3,2)
Transferência de outro estado	5.471 (1,9)
Transferência de outro país	166 (0,1)
Recidiva	13.473 (4,6)
Outros reingressos	24.307 (8,3)
Ignorado	404 (0,1)
Modo de detecção de caso novo	
Encaminhamento	107.949 (45,6)
Demanda espontânea	93.154 (39,3)
Exame de coletividade	9.386 (4,0)
Exame de contatos	20.396 (8,6)
Outros modos	5.191 (2,2)
Ignorado	818 (0,3)
Baciloscopia	
Positiva	55.369 (27,2)
Não realizada	67.281 (33,0)
Negativa	74.700 (36,7)
Ignorado	6.408 (3,1)
Esquema terapêutico inicial	
Poliquimioterapia/paucibacilar/6 doses	63.256 (21,6)
Poliquimioterapia/multibacilar/12 doses	223.065 (76,3)
Outros esquemas substitutos	5.931 (2,1)
Episódio reacional durante o tratamento	
Tipo 1	27.210 (12,5)
Tipo 2	8.887 (4,1)
Tipo 1 e 2	3.964 (1,8)
Sem informação	177.254 (81,6)

**Tabela 2:** Indicadores operacionais da hanseníase associados ao grau de incapacidade. Brasil, 2014-2023 (n=228.577)

Indicador	2014	2015	2016	2017	2018	2019	2020	2021	2022	2023^a^	Total
Proporção de casos novos de hanseníase com grau de incapacidade física avaliado no momento do diagnóstico											
Norte	92,8	92,3	92,8	94,1	94,5	93,8	92,9	94,3	92,4	93,0	93,3
Nordeste	84,9	85,3	84,9	84,0	83,9	83,3	82,9	83,6	81,7	83,7	84,0
Sudeste	94,4	93,6	93,3	92,2	92,8	91,0	91,3	90,3	88,2	84,0	91,9
Sul	94,6	95,1	95,1	94,9	93,8	93,7	87,6	91,2	88,2	83,4	92,9
Centro-Oeste	88,4	88,6	88,2	89,8	86,9	88,0	83,6	84,5	83,9	82,0	87,1
Brasil	88,8	88,7	88,6	88,6	88,1	87,7	86,2	87,0	85,1	85,0	87,8
Proporção de casos novos de hanseníase com incapacidade física grau 2 no momento do diagnóstico											
Norte	6,7	6,7	7,4	8,4	8,7	9,4	8,8	12,3	11,5	12,0	8,6
Nordeste	6,8	7,0	6,9	7,8	8,1	8,5	8,7	9,9	9,6	9,9	8,0
Sudeste	11,2	10,6	13,5	11,9	11,6	15,0	14,9	14,5	16,1	17,9	13,1
Sul	10,4	10,1	11,8	13,3	14,4	15,0	12,2	13,9	15,1	13,7	12,7
Centro-Oeste	6,8	6,3	6,6	6,0	6,0	8,8	9,7	9,7	11,0	11,5	7,6
Brasil	7,6	7,4	8,1	8,3	8,4	9,8	10,0	11,2	11,5	12,0	9,0
Taxa de incidência de hanseníase com incapacidade física grau 2 no momento do diagnóstico por 1 milhão de habitantes											
Norte	22,13	18,79	19,39	22,64	26,23	25,07	14,30	20,36	17,87	5,53	19,12
Nordeste	14,31	13,81	11,54	13,80	14,18	14,51	9,60	11,53	12,03	3,09	11,81
Sudeste	5,70	4,77	5,48	4,76	4,50	5,74	3,98	4,31	4,91	1,40	4,54
Sul	3,54	3,37	3,17	3,39	3,60	3,80	2,05	2,70	2,81	0,71	2,90
Centro-Oeste	24,45	20,46	17,75	18,34	21,70	30,86	19,21	16,88	20,64	6,78	19,61
Brasil	10,57	9,45	8,95	9,63	10,23	11,49	7,32	8,44	8,92	2,55	8,72
Proporção de casos novos de hanseníase, na população de 0-14 anos, com incapacidade física grau 2 no momento do diagnóstico											
Norte	2,9	2,8	3,8	3,4	3,1	4,4	5,1	5,9	6,3	3,8	3,7
Nordeste	2,4	2,6	1,6	3,1	2,8	3,6	4,1	3,7	5,6	6,2	3,0
Sudeste	1,1	6,1	1,9	3,0	-	1,5	3,8	2,3	10,7	4,5	3,0
Sul	-	-	16,7	10,5	10,0	-	-	-	-	-	3,5
Centro-Oeste	2,0	1,9	1,9	2,9	2,7	4,1	7,4	3,2	3,1	-	2,8
Brasil	2,3	2,8	2,3	3,2	2,6	3,6	4,7	4,0	5,9	4,0	3,2

a Dados referentes aos meses de janeiro a maio.

**Tabela 3:** Variação percentual anual (VPA) e intervalos de confiança (IC95%) dos indicadores operacionais da hanseníase associados ao grau de incapacidade. Brasil, 2014-2023 (n=228.577)

Variáveis	VPA (IC95%)	p-valor	D-W^a^	Interpretação
Proporção de casos novos de hanseníase com grau de incapacidade física avaliado no momento do diagnóstico				
Norte	0,0 (-0,2; 0,3)	0,711	1,921	Estacionária
Nordeste	-0,4 (-0,5; -0,3)	<0,001	1,925	Diminuição
Sudeste	-1,2 (-1,8; -0,5)	0,003	1,184	Diminuição
Sul	-1,3 (-1,8; -0,7)	<0,001	1,619	Diminuição
Centro-Oeste	-0,9 (-1,3; -0,5)	<0,001	1,723	Diminuição
Brasil	-0,5 (-0,7; -0,4)	<0,001	1,763	Diminuição
Proporção de casos novos de hanseníase com incapacidade física grau 2 no momento do diagnóstico				
Norte	7,5 (6,3; 8,8)	<0,001	2,448	Crescimento
Nordeste	4,9 (4,1; 5,7)	<0,001	2,066	Crescimento
Sudeste	5,2 (3,7; 6,8)	<0,001	2,442	Crescimento
Sul	3,5 (0,4; 6,7)	0,032	1,680	Crescimento
Centro-Oeste	7,3 (2,9; 11,8)	0,005	1,615	Crescimento
Brasil	6,0 (5,1; 6,9)	<0,001	1,679	Crescimento
Taxa de incidência de hanseníase com incapacidade física grau 2 no momento do diagnóstico por 1 milhão de habitantes				
Norte	-1,8 (-7,2; 3,9)	0,467	2,007	Estacionária
Nordeste	-2,6 (-6,0; 0,9)	0,121	2,120	Estacionária
Sudeste	-2,4 (-4,3; -0,3)	0,028	2,791	Diminuição
Sul	-3,9 (-8,3; 0,6)	0,080	2,104	Estacionária
Centro-Oeste	-1,0 (-7,0; 5,4)	0,713	1,856	Estacionária
Brasil	-2,0 (-5,4; 1,5)	0,210	2,098	Estacionária
Proporção de casos novos de hanseníase, na população de 0-14 anos, com incapacidade física grau 2 no momento do diagnóstico				
Norte	7,9 (3,1; 13,0)	0,005	1,680	Crescimento
Nordeste	12,5 (8,4; 16,7)	<0,001	2,064	Crescimento
Sudeste	10,0 (-1,6; 23,0)	0,083	1,519	Estacionária
Sul^b^	-	-	-	-
Centro-Oeste	11,0 (-0,6; 24,0)	0,061	1,797	Estacionária
Brasil	9,9 (7,5; 12,5)	<0,001	1,933	Crescimento

a D-W: Durbin-Watson; ^b^ Dados insuficientes para análise.

**Tabela 4:** Razões de chances (*odds ratio*, OR) brutas e ajustadas e intervalos de confiança de 95% (IC95%) do grau de incapacidade 1 da hanseníase pelas variáveis sociodemográficas e clínicas. Brasil, 2014-2023 (n=228.577)

Preditores	OR bruta (IC95%)	p-valor	OR ajustada (IC95%)	p-valor
Faixa etária (anos)				
60+	4,17 (3,96; 4,38)	<0,001	3,36 (3,15; 3,58)	<0,001
15-19	1,64 (1,53; 1,75)	<0,001	1,64 (1,51; 1,79)	<0,001
20-39	2,37 (2,25; 2,49)	<0,001	2,04 (1,91; 2,18)	<0,001
40-59	3,30 (3,14; 3,47)	<0,001	2,62 (2,47; 2,79)	<0,001
0-14	1,00		1,00	
Sexo				
Masculino	1,32 (1,30; 1,34)	<0,001	0,97 (0,64; 1,49)	0,879
Feminino	1,00		1,00	
Gestante				
Sim	1,07 (0,61; 1,89)	0,747	1,32 (1,13; 1,54)	0,001
Não	1,00		1,00	
Raça/cor da pele				
Preta	1,00 (0,97; 1,03)	0,787	1,03 (0,99; 1,07)	0,098
Parda	0,89 (0,87; 0,91)	<0,001	0,95 (0,93; 0,98)	0,001
Amarela	1,05 (0,96; 1,15)	0,265	0,99 (0,89; 1,11)	0,907
Indígena	1,01 (0,88; 1,15)	0,908	1,17 (0,99; 1,38)	0,070
Branca	1,00		1,00	
Escolaridade				
Analfabeto	2,00 (1,91; 2,10)	<0,001	1,50 (1,41; 1,61)	<0,001
Ensino fundamental	1,44 (1,38; 1,51)	<0,001	1,36 (1,29; 1,44)	<0,001
Ensino médio	1,11 (1,06; 1,16)	<0,001	1,15 (1,09; 1,22)	<0,001
Ensino superior	1,00		1,00	
Forma clínica				
Indeterminada	0,37 (0,35; 0,39)	<0,001	0,82 (0,76; 0,88)	<0,001
Tuberculoide	0,44 (0,42; 0,46)	<0,001	0,95 (0,89; 1,02)	0,131
Dimorfa	1,46 (1,40; 1,52)	<0,001	1,11 (1,06; 1,17)	<0,001
Virchowiana	1,94 (1,86; 2,03)	<0,001	1,43 (1,35; 1,52)	<0,001
Não classificado	1,00		1,00	
Classificação operacional				
Multibacilar	4,55 (4,43; 4,67)	<0,001	2,05 (1,94; 2,16)	<0,001
Paucibacilar	1,00		1,00	
Tipo de entrada				
Transferência	1,80 (1,75; 1,85)	<0,001	1,39 (1,28; 1,51)	<0,001
Recidiva	1,52 (1,48; 1,58)	<0,001	1,52 (1,36; 1,69)	<0,001
Caso novo	1,00		1,00	
Modo de detecção de caso novo				
Encaminhamento	0,80 (0,75; 0,86)	<0,001	0,95 (0,88; 1,02)	0,171
Demanda espontânea	0,79 (0,74; 0,85)	<0,001	0,91 (0,84; 0,99)	0,020
Exame de coletividade	0,92 (0,85; 1,00)	0,059	1,16 (1,06; 1,27)	0,002
Exame de contatos	0,79 (0,73; 0,85)	<0,001	0,87 (0,80; 0,95)	0,002
Outros modos	1,00		1,00	
Baciloscopia				
Positiva	1,56 (1,53; 1,60)	<0,001	1,09 (1,05; 1,12)	<0,001
Não realizada	1,05 (1,03; 1,08)	<0,001	0,91 (0,88; 0,94)	<0,001
Negativa	1,00		1,00	
Número de nervos afetados	1,49 (1,48; 1,50)	<0,001	1,41 (1,40; 1,42)	<0,001

**Tabela 5:** Razões de chances (*odds ratio*, OR) brutas e ajustadas e intervalos de confiança de 95% (IC95%) do grau de incapacidade 2 da hanseníase pelas variáveis sociodemográficas e clínicas. Brasil, 2014-2023 (n=228.577)

Preditores	OR bruta (IC95%)	p-valor	OR ajustada (IC95%)	p-valor
Faixa etária (anos)				
60+	7,04 (6,43; 7,72)	<0,001	4,10 (3,65; 4,59)	<0,001
15-19	1,95 (1,74; 2,20)	<0,001	1,85 (1,61; 2,13)	<0,001
20-39	2,90 (2,65; 3,18)	<0,001	2,08 (1,86; 2,33)	<0,001
40-59	4,17 (3,80; 4,56)	<0,001	2,58 (2,31; 2,89)	<0,001
0-14	1,00		1,00	
Sexo				
Masculino	2,19 (2,13; 2,25)	<0,001	1,56 (1,51; 1,62)	<0,001
Feminino	1,00		1,00	
Gestante				
Sim	0,54 (0,43; 0,69)	<0,001	1,11 (0,86; 1,42)	0,420
Não	1,00		1,00	
Raça/cor da pele				
Preta	1,05 (1,00; 1,10)	0,032	1,01 (0,96; 1,07)	0,595
Parda	0,86 (0,83; 0,89)	<0,001	0,93 (0,89; 0,96)	<0,001
Amarela	0,85 (0,74; 0,98)	0,025	0,79 (0,66; 0,94)	0,007
Indígena	1,06 (0,88; 1,29)	0,516	1,20 (0,96; 1,49)	0,117
Branca	1,00		1,00	
Escolaridade				
Analfabeto	4,21 (3,84; 4,63)	<0,001	2,80 (2,50; 3,13)	<0,001
Ensino fundamental	2,35 (2,17; 2,53)	<0,001	2,07 (1,88; 2,27)	<0,001
Ensino médio	1,39 (1,27; 1,51)	<0,001	1,44 (1,30; 1,59)	<0,001
Ensino superior	1,00		1,00	
Forma clínica				
Indeterminada	0,12 (0,11; 0,14)	<0,001	0,39 (0,34; 0,44)	<0,001
Tuberculoide	0,23 (0,21; 0,25)	<0,001	0,71 (0,63; 0,79)	<0,001
Dimorfa	1,10 (1,04; 1,17)	0,001	0,75 (0,70; 0,81)	<0,001
Virchowiana	2,42 (2,27; 2,57)	<0,001	1,43 (1,04; 1,33)	<0,001
Não classificado	1,00		1,00	
Classificação operacional				
Multibacilar	11,41 (11,05; 11,78)	<0,001	1,99 (1,54; 2,59)	<0,001
Paucibacilar	1,00		1,00	
Tipo de entrada				
Transferencia	2,79 (2,69; 2,88)	<0,001	1,77 (1,68; 1,86)	<0,001
Recidiva	1,70 (1,62; 1,78)	<0,001	1,34 (1,26; 1,42)	<0,001
Caso novo	1,00		1,00	
Modo de detecção de caso novo				
Encaminhamento	0,45 (0,42; 0,49)	<0,001	0,85 (0,74; 0,96)	0,016
Demanda espontânea	0,35 (0,32; 0,38)	<0,001	0,60 (0,52; 0,70)	<0,001
Exame de coletividade	0,35 (0,31; 0,39)	<0,001	0,70 (0,58; 0,85)	0,002
Exame de contatos	0,23 (0,21; 0,26)	<0,001	0,41 (0,35; 0,49)	<0,001
Outros modos	1,00		1,00	
Baciloscopia				
Positiva	1,85 (1,76; 1,94)	<0,001	0,92 (0,86; 0,99)	0,024
Não realizada	0,92 (0,88; 0,96)	<0,001	0,77 (0,73; 0,81)	<0,001
Negativa	1,00		1,00	
Esquema terapêutico inicial				
Poliquimioterapia/paucibacilar/6 doses	0,05 (0,04; 0,05)	<0,001	0,56 (0,43; 0,73)	<0,001
Poliquimioterapia/multibacilar/12 doses	0,54 (0,50; 0,58)	<0,001	0,86 (0,77; 0,96)	0,007
Outros esquemas substitutos	1,00		1,00	
Número de nervos afetados	1,61 (1,59; 1,62)	<0,001	1,53 (1,52; 1,55)	<0,001

## Discussão 

A avaliação dos dados epidemiológicos apontou o total de 228.577 casos diagnosticados de hanseníase nas regiões do Brasil no período analisado. Os resultados indicaram menores chances de apresentar incapacidades físicas de grau 1 ou grau 2 associadas ao sexo feminino, à forma clínica tuberculoide, à residência nas regiões Sudeste e Sul e à condição de nível superior. Ademais, casos novos de hanseníase em indivíduos na faixa etária de 40-59 anos, menor nível de escolaridade e forma clínica multibacilar apresentaram maiores chances de desenvolver incapacidades físicas de grau 1 ou 2 no momento do diagnóstico.

O estudo apresenta limitações relacionadas aos dados faltantes, especialmente sobre a avaliação da incapacidade no diagnóstico. A ausência dessas informações impede uma análise mais completa e pode induzir viés, caso as perdas de dados não sejam aleatórias. Para lidar com isso, foi adotado o procedimento de imputação múltipla, assumindo que as perdas são aleatórias (*missing at random*), o que é comum em estudos dessa natureza. Além disso, a qualidade dos dados, proveniente de atividades de vigilância de rotina, nem sempre garante a coleta consistente de todas as informações relevantes, como características dos serviços de saúde e percepção dos pacientes, limitando a análise. Apesar dessas limitações, a imputação múltipla ajudou a minimizar o impacto dos dados faltantes, mas melhorias na coleta e na análise de dados são necessárias em estudos futuros.

Este estudo se destaca por preencher uma lacuna na literatura ao explorar a relação entre os graus de incapacidade física da hanseníase e os fatores associados com abordagem abrangente e representatividade nacional. Ele utiliza dados recentes e investiga a distribuição espacial e temporal da doença, permitindo identificar padrões geográficos influenciados por determinantes sociais e ambientais.

Indivíduos do sexo masculino apresentaram predominância em comparação ao feminino. Os dados indicaram que homens apresentam maior exposição ao *Mycobacterium leprae*, possivelmente devido à menor atenção aos cuidados com a saúde, influenciados por fatores culturais. Essa negligência pode resultar em atrasos no diagnóstico, aumentando o risco de desenvolvimento de incapacidades físicas. Estudos mostram que populações masculinas enfrentam barreiras programáticas nos serviços de saúde, o que intensifica a vulnerabilidade ao diagnóstico tardio e às complicações da hanseníase ^17^ ^,^ ^18^.

Neste estudo, as regiões Sul e Sudeste apresentaram as maiores proporções de casos novos com incapacidade física 2. Essa proporção é alta se se considerar o parâmetro do Ministério da Saúde de <5,0% para baixo, de 5,0%-9,9% para médio e de >10,0% para alto ^8^. Não é possível justificar com precisão o motivo desse cenário nessas regiões, porém, uma maior concentração de grau 2 de incapacidade entre menores de 15 anos pode ajudar a explicar esse padrão. 

Nos últimos anos, diversos marcos surgiram com o foco de reduzir os casos nessa população, como: a estratégia global da hanseníase (2016-2020); a implementação da vigilância da incapacidade física de grau 2 em menores de 15 anos no Brasil em 2018; a estratégia global para hanseníase (2021-2030); e a implementação da nota N1/2023, que recomenda e orienta a implementação da vigilância do grau 2 para todos os casos novos ^19^. 

Dessa forma, essa alta proporção mostra-se preocupante, indicando a necessidade de ajustes nas estratégias relacionadas ao diagnóstico precoce e ao tratamento, visando minimizar o impacto das incapacidades associadas à hanseníase ^20^. É importante aprimorar a avaliação neurológica simplificada e utilizá-la principalmente como ferramenta impulsionadora do cuidado às pessoas afetadas pela hanseníase, tanto durante quanto após o tratamento com poliquimioterapia. Torna-se igualmente necessário reforçar ações de busca ativa para identificar precocemente casos novos, investigar contatos próximos e implementar medidas preventivas adicionais, tais como a quimioprofilaxia, visando reduzir a transmissão e prevenir o surgimento de novos casos incapacitantes.

A menor probabilidade de graus 1 e 2 de incapacidade física foi observado nos casos residentes nas regiões Sudeste e Sul. Os dados indicaram maiores proporções dessas incapacidades em municípios com altas taxas de incidência de hanseníase, predominantemente localizados nas regiões Norte e Nordeste. Estes achados são consistentes com estudos que destacam a relação entre a alta carga da doença e a maior prevalência de formas incapacitantes em áreas com menos acesso a diagnóstico precoce e a tratamento adequado ^21^ ^,^ ^22^ ^,^ ^23^. 

As regiões Sudeste e Sul do Brasil se destacam por apresentarem índices socioeconômicos mais elevados, o que proporciona uma infraestrutura mais robusta em termos de saúde e serviços essenciais ^24^. Essa realidade distingue-se com as regiões Norte, Nordeste e Centro-Oeste, que historicamente enfrentam desafios socioeconômicos significativos, como a falta de recursos e de acesso a serviços de saúde de qualidade. Assim, a menor prevalência de hanseníase observada na região Sul reflete, em parte, o maior nível de desenvolvimento dessas áreas ^25^ ^,^ ^26^. O acesso mais amplo à informação, à prevenção e ao tratamento adequado favorece a redução dos casos, enquanto as regiões com menores índices de desenvolvimento enfrentam dificuldades que agravam a situação da doença, incluindo diagnósticos mais tardios e maior prevalência de formas incapacitantes.

Este estudo indica que níveis mais elevados de escolaridade estão menos associados à presença de deficiências físicas no diagnóstico, o que corrobora os estudos existentes ^27^ ^,^ ^28^. utro estudo ^29^ aponta que, quanto menor o nível de escolaridade, maior a vulnerabilidade e a negligência, mantendo a hanseníase como um problema que acompanha os diferentes ciclos da vida ^29^. Um maior nível educacional está relacionado a uma compreensão aprimorada da condição de saúde e, por consequência, a um melhor acesso e utilização dos serviços de saúde. A realização de avaliações periódicas e a prática de autocuidado são fatores que podem auxiliar na prevenção do agravamento dos sintomas clínicos.

A maior probabilidade de incapacidades relacionadas à hanseníase foi observada em indivíduos com mais de 40 anos. Essa faixa etária, frequentemente ativa no mercado de trabalho, pode enfrentar limitações ocupacionais devido às incapacidades provocadas pela doença. Tais restrições podem resultar em afastamentos, aposentadorias precoces e uma redução significativa na qualidade de vida, impactando tanto os trabalhadores quanto as famílias ^30^. Nesse contexto, a implementação de ações voltadas à redução das sequelas físicas e à reintegração desses indivíduos no ambiente de trabalho seria uma medida transformadora, visto que a reintegração ocupacional possibilita que as pessoas afetadas pela hanseníase possam retomar as atividades profissionais, reduzindo a perda de renda e a exclusão social.

A hanseníase na forma multibacilar está associada a um maior risco de desenvolvimento de incapacidades físicas de grau 1 ou 2 no momento do diagnóstico. Estudos conduzidos em municípios brasileiros evidenciam que a classificação operacional da doença possui relação estatisticamente significativa com a ocorrência dessas incapacidades, apresentando um risco 99,0% maior de desenvolver sequelas em comparação aos paucibacilares. A transmissão contínua em áreas com acesso limitado a cuidados de saúde contribui para um maior número de casos, que possuem um risco elevado de evolução para formas incapacitantes. O que ressalta a importância do diagnóstico precoce, bem como a implementação de estratégias específicas para identificar e tratar esses casos, com o objetivo de reduzir o risco de incapacidades físicas e melhorar os desfechos para os pacientes ^31^ ^,^ ^17^.

Em conclusão, os achados revelam que mesmo a hanseníase sendo uma das doenças mais antigas já relatadas, os avanços no diagnóstico e no tratamento são de extrema importância, além da educação em saúde e das políticas públicas inclusivas aplicadas de forma disciplinada, que continuam sendo necessárias para que haja redução nos casos das regiões de maior endemia. A distribuição geográfica e temporal da hanseníase reflete as desigualdades no acesso ao diagnóstico e ao tratamento, com destaque para as regiões economicamente mais vulneráveis. O desafio é não apenas aumentar a detecção precoce, mas também garantir que os cuidados de saúde cheguem a todos, de maneira igualitária, e que a sociedade compreenda a importância da prevenção, do tratamento contínuo e da integração dos pacientes na sociedade.

## Data Availability

O banco de dados e a descrição das variáveis estão disponíveis em: https://doi.org/10.48331/scielodata.UOZXWF.
